# Single Injection of Cross-Linked Hyaluronate in Knee Osteoarthritis: A 52-Week Double-Blind Randomized Controlled Trial

**DOI:** 10.3390/pharmaceutics14091783

**Published:** 2022-08-25

**Authors:** Po-Yen Ko, Chung-Yi Li, Chia-Lung Li, Li-Chieh Kuo, Wei-Ren Su, I-Ming Jou, Po-Ting Wu

**Affiliations:** 1 Department of Orthopedics, National Cheng Kung University Hospital, College of Medicine, National Cheng Kung University, Tainan 704302, Taiwan; 2 Department of Biomedical Engineering, National Cheng Kung University, Tainan 701401, Taiwan; 3 Department of Public Health, College of Medicine, National Cheng Kung University, Tainan 701401, Taiwan; 4 Department of Public Health, College of Public Health, China Medical University, Taichung 404328, Taiwan; 5 Department of Healthcare Administration, College of Medical and Health Science, Asia University, Taichung 413305, Taiwan; 6 Department of Orthopedics, Tainan Hospital, Ministry of Health and Welfare, Tainan 700043, Taiwan; 7 Department of Occupational Therapy, College of Medicine, National Cheng Kung University, Tainan 701401, Taiwan; 8 Medical Device Innovation Center, National Cheng Kung University, Tainan 701401, Taiwan; 9 Department of Orthopedics, College of Medicine, National Cheng Kung University, Tainan 701401, Taiwan; 10 Department of Orthopedics, E-Da Hospital, Kaohsiung 824005, Taiwan; 11 School of Medicine, College of Medicine, I-Shou University, Kaohsiung 824005, Taiwan; 12 GEG Orthopedic Clinic, Tainan 701002, Taiwan; 13 Department of Biochemistry and Molecular Biology, College of Medicine, National Cheng Kung University, Tainan 701401, Taiwan

**Keywords:** osteoarthritis, cross-linked hyaluronate, knee, viscosupplementation

## Abstract

Background: to compare the 52-week effectiveness and safety between HYAJOINT Plus (HJP) and Durolane in knee osteoarthritis (OA) treatment. Methods: consecutive patients received a single injection of 3 mL HJP or Durolane. The primary outcome was a visual analog scale (VAS) pain measurement at 26 weeks post-injection. Secondary outcomes included other clinical, satisfaction, and safety assessments for 52 weeks. Results: 142 patients were equally randomized. At week 26, the HJP group had less VAS pain than the Durolane group (18.1 ± 9.5 versus 24.4 ± 14.0, *p* = 0.001). Both groups showed improvement in their VAS pain and stiffness scores, and Western Ontario and McMaster Universities Osteoarthritis Index (WOMAC) pain and total scores for 52 weeks after injection (*p* < 0.001). However, the HJP group showed lower VAS pain and stiffness scores, reduced WOMAC pain and stiffness scores, a shorter Timed “Up & Go” (TUG) time, and a higher satisfaction score than the Durolane group for 39 weeks (*p* < 0.05). Only mild and self-limited adverse events occurred (40.8%). Conclusion: While a single injection of either HJP or Durolane is safe and effective for at least 52 weeks, HJP provided superior improvement in terms of VAS pain and stiffness scores, WOMAC pain and stiffness scores, and satisfaction score within 39 weeks of treatment.

## 1. Introduction

Osteoarthritis (OA) is the world’s fastest increasing major health condition [1], and it mainly affects the knee joint [2]. Despite the ongoing debate in numerous meta-analyses [3,4,5] regarding the efficacy of intra-articular hyaluronate (IAHA), exogenous IAHA remains widely used in clinical practice, especially for knee OA [6,7]. A recent meta-analysis [8] showed that the most effective and safe HA products were those that derive HA from biological fermentation and have a molecular weight ≥ 3000 kDa.

Numerous trials have reported that cross-linked HA (cHA) can relieve knee pain for up to 26 weeks after injection [9,10,11]. However, the different cross-linking techniques might lead to different levels of effectiveness [11]. Even though both HYAJOINT Plus (HJP; SciVision Biotech, Kaohsiung, Taiwan) and Durolane (Bioventus LLC, Durham, NC, USA) are produced by biofermentation and cross-linked by 1,4-butanediol diglycidyl ether (BDDE), HJP is synthesized in a novel cross-linking technique (crosslinked hyaluronic acid platform, CHAP, Supplementary Figure S1). In response, we performed a randomized, controlled, double-blind trial to compare the 52-week effectiveness and safety between HJP and Durolane in knee OA. We hypothesized that HJP would lead to better visual analog scale (VAS) pain relief than Durolane at week 26.

## 2. Materials and Methods

### 2.1. Ethic Statement

The study was performed in a university-affiliated medical center in accordance with Good Clinical Practice (GCP) principles from June 2017 to July 2019 and was approved by the institutional review board of the authors’ hospital (A-BR-105-090). In addition, this study was registered at Clinicaltrials.gov (NCT04000204), and all participants provided written informed consent prior to enrollment.

### 2.2. Participants

A total of 151 consecutive patients with primary knee OA, as defined by the American College of Rheumatology (ACR) criteria [12], were recruited for eligibility screening. The inclusion and exclusion criteria are listed in Table 1. After a screening visit, all eligible patients returned for their baseline visit after a 1-week period to allow for the washout of any ingested NSAIDs or analgesics and received the single IAHA injection into the suprapatellar pouch under ultrasound (US)-guidance [13]. At one week post-injection, we contacted the participants via telephone to collect data related to the safety of the injection. Follow-up visits were arranged for functional outcomes and safety assessments at 4, 12, 26, 39, and 52 weeks post-injection.

### 2.3. Randomization and Treatments

Enrolled patients were randomized into two groups with equal numbers. For randomization, sequentially numbered envelopes, in which the allocation was sealed, were generated by an assistant coordinator who was not clinically involved in the study using a random number table calculated online. According to the allocation in the envelope, the patient was given the allocated cHA, 3-mL HJP (20 mg/mL) or 3-mL Durolane (20 mg/mL). The two cHA products were prepared in a similar syringe without any marking that could provide identifying information. All IAHA injections were performed by the principal investigator (PTW). If there was any evidence of ultrasonographic suprapatellar effusion (SPE) before injection, complete effusion aspiration was done under US-guidance to prevent dilution of the cHA. Another investigator (co-investigator, CLL) who was blinded to the randomization and treatment performed all assessments. The patients were also blinded to the treatment during the study period and injection process. No regular analgesics, glucosamine, chondroitin or NSAIDs were permitted during the study. Use of acetaminophen (maximum daily dose: 4 g) as the only rescue medication and aspirin (maximum daily dose: 325 mg) as an anti-coagulation therapy was allowed. Acetaminophen was not permitted within 48 h prior to the follow-up visit and was recorded when needed for rescue by the patient in a diary. Major protocol violations included initiation of physical therapy, use of prescribed medication, and surgery.

### 2.4. Functional Outcomes and Safety Assessmens

The primary outcome was VAS pain measurements at 26 weeks post-injection. Secondary outcomes included the Western Ontario and McMaster Universities Osteoarthritis Index (WOMAC) [14], the Timed “Up & Go” (TUG) test [15], the single-limb stance (SLS) test [16], patient satisfaction (0–100 mm), US parameters, and safety assessments for the next 52 weeks. All assessments were evaluated at each follow-up visit.

For ultrasound assessment, both longitudinal and transverse scans were conducted using a Logiq-e R7scanner (GE Healthcare, Madison, WI, USA) with a L4-12t-RS (4.2–13.0 MHz) linear array transducer. The US examination was performed on the same day by two operators blinded to the patients’ clinical information. The inter-observer agreement for each US parameter, based on previous studies [17,18], was evaluated using the κ statistic; the results were 0.84 for suprapatellar synovitis, 0.86 for SPE, 0.84 for medial compartment synovitis, and 0.86 for lateral compartment synovitis (LCS). Discrepancies in US inflammatory characteristics were resolved by consensus.

Safety was assessed according to adverse events reported by patients and physical findings by the co-investigator (CLL) at each visit. A serious adverse event was defined as any event leading to hospitalization, permanent disability, or other life-threatening condition. The severity and causality of the adverse event were determined by the co-investigator (CLL).

### 2.5. Statistical Analysis

The SPSS SamplePower 3.0 software (IBM) was used to estimate the required sample size based on the independent samples 1-way analysis of covariance (ANCOVA) using baseline data as the covariates. Since there was no prior data from comparing the two products using ANCOVA, an R^2^ medium-level Cohen effect size of 0.09 for the covariates and a medium-level effect size of 0.25 for ANOVA were chosen as the desired effect size. Fifty-nine participants per group were required to achieve a statistical power of 0.8 at an alpha level of 0.05. Further assuming a 15% dropout rate, the number of participants needed to be at least 70 per group.

The difference in main outcome (i.e., VAS at the 26th week) was assessed by an intention-to-treat (ITT) analysis, in which patients were analyzed according to the treatment initially assigned. Demographic and baseline data were compared using the two independent *t*-tests for continuous variables and the Chi-square test for categorical variables. Further exploratory analyses included between-group comparisons of various outcomes with independent *t*-tests at various points in time during the 52-week follow-up, as well as within-group comparisons using a linear regression model with the generalized estimating equation (GEE) method to accommodate within-subject correlation [19]. Significance was set at *p* < 0.05. Data were analyzed using SAS 9.3 for Windows (SAS Institute, Cary, NC, USA).

## 3. Results

### 3.1. Demographic Data

**Figure 1:**
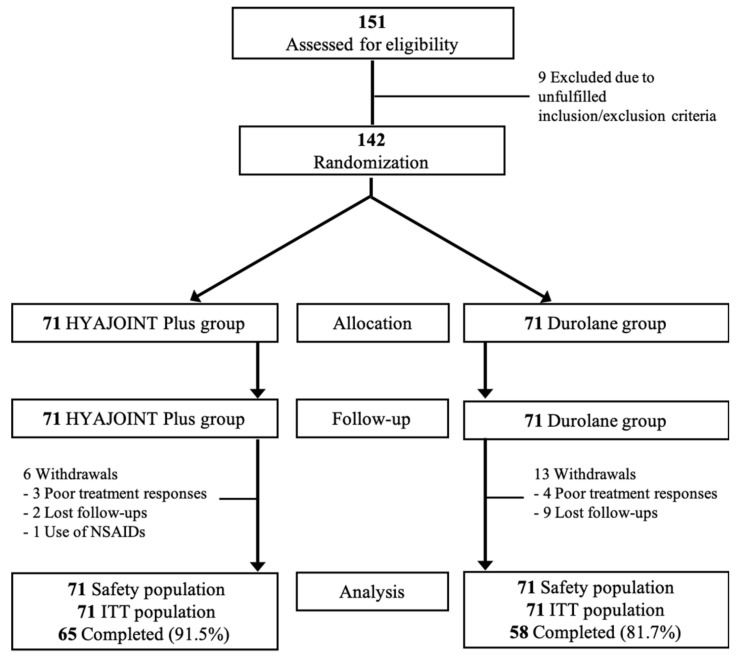
Flow diagram of the randomized controlled trial.

Ultimately, a total of 142 participants were randomized into either the HJP group (*n* = 71) or Durolane group (*n* = 71) (Figure 1). Nineteen patients withdrew from this study, with 7 cases reporting this to be due to poor treatment response, 11 cases due to loss of follow-up, and 1 case due to protocol violation with use of NSAIDs. All 142 patients were available for ITT and safety analyses. There were no significant differences in demographic and baseline data between the two groups, except for the proportion of inflammation in LCS (Table 2).

### 3.2. Clinical Outcomes

**Figure 2:**
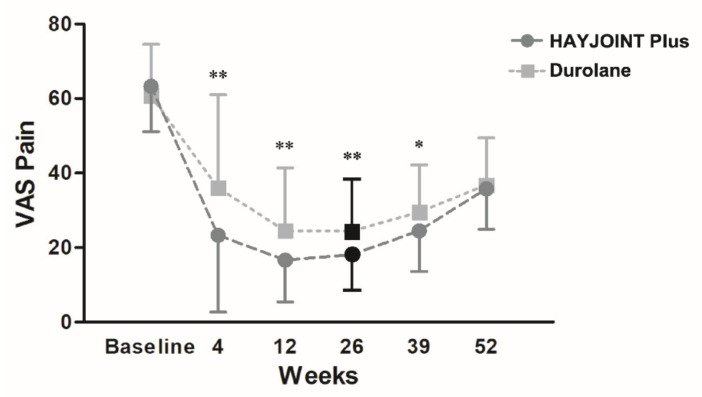
Changes in visual analog score (VAS) pain throughout the trial. The between-group difference * *p* < 0.05, ** *p* < 0.01.

For the primary outcome, at week 26, the mean VAS pain score significantly decreased from 63.3 ± 12.2 at baseline to 18.1 ± 9.5 in the HJP group, and 60.8 ± 13.8 at baseline to 24.4 ± 14.0 in the Durolane group (both *p* < 0.01). The VAS pain score was significantly less in the HJP group (*p* = 0.001, Figure 2). For secondary outcomes, both groups showed significant improvement in their VAS pain score, VAS stiffness score, WOMAC pain score, WOMAC function score, and WOMAC total score four weeks after injection (all *p* < 0.001) in comparison to the baseline. These improvements, except in the WOMAC function score, significantly persisted for 52 weeks (all *p* < 0.001, Table 3). For the WOMAC stiffness score, the improvement in the HJP group remained significant until 52 weeks, but was not significant in the Durolane group throughout the study. The HJP group consistently showed a lower VAS pain score, VAS stiffness score (except at week 4), and WOMAC pain and stiffness scores (except at week 4) than the Durolane group from weeks 4 to 39 post injection (*p* < 0.01), and a lower WOMAC total score at weeks 12 and 26 (*p* < 0.05).

For the TUG test, the HJP group recorded a significantly shorter time than the Durolane group until week 52 (*p* < 0.05, Table 3). In spite of there being no between-group difference in SLS time throughout the entire study, the improvement in the SLS test was significant in the HJP group from weeks 4 to 52 (*p* < 0.05), but was not significant in the Durolane group until week 26. Therefore, there was a significant interaction of study group and time in the SLS test that suggested a more evident improvement trend in the HJP group. On the other hand, the lack of significant interaction of study group with time for all other clinical parameters except the SLS test and the WOMAC stiffness score over the 52-week period suggests a similarity in linear decreasing trend in both groups (Table 3).

In both groups, there was no acetaminophen consumption for knee discomfort. The satisfaction score was significantly higher in the HJP group from weeks 4 to 39 (*p* < 0.01, Table 4), and reached a peak at 26 weeks.

### 3.3. US Outcomes

The patients with US inflammatory features at baseline visit were included for subgroup analyses. In these patients, the percentage of inflammation monotonically and significantly decreased in both groups during the follow-ups (*p* < 0.05, Supplementary Table S1).

### 3.4. Safety Outcomes

The incidence and type of adverse events are listed in Table 5. The overall rate was 40.8%, and rates were comparable between the two groups. All adverse events related to the study treatment occurred following the IAHA injection; however, they were considered of mild grade and resolved spontaneously within one week. No incidences of superficial infections or septic joints, and no allergic reactions, systemic reactions, or serious adverse events occurred during the study period. Only one infection event unrelated to the study treatment was reported. No adverse event led to a study cessation in either group.

## 4. Discussion

This study examined the effectiveness and safety of HJP and Durolane for knee OA over a 52-week period. In the primary outcome, the HJP group had significantly lower VAS pain than the Durolane group at week 26. Furthermore, the HJP group revealed a better VAS pain and stiffness score, WOMAC pain and stiffness scores, and satisfaction score than the Durolane group for 39 weeks. Only mild and self-limited adverse events occurred during the trial period, with an overall rate of 40.8% following the injections. Our results show that a single injection of either HJP or Durolane can be considered safe and significantly effective for knee OA for 52 weeks.

Altman et al. [8] reported in a meta-analysis that biologically fermented HA with a molecular weight ≧3000 kDa had superior efficacy and safety. Both HJP and Durolane are biofermented cHA products with molecular weights greater than 3000 kDa. Both products contain the same volume of HA (3 mL) with the same concentration (20 mg/mL) and use the same cross-linking agent, 1,4,-BDDE. However, Durolane is synthesized using the non-animal stabilized hyaluronic acid (NASHA) cross-linking technique, and HJP using the CHAP technique (Supplementary Figure S1). These two different techniques also lead to the gels’ different appearance. Durolane gel is granular-texture type, while HJP is more gel-like type. With respect to the between-group differences, our primary outcome revealed the HJP group had significantly lower VAS pain scores than the Durolane group at week 26. Furthermore, the HJP group showed lower VAS pain and stiffness scores, and WOMAC pain and stiffness scores within the first 39 weeks compared to the Durolane group. Correspondingly, the HJP group reported a higher satisfaction score for 39 weeks. The difference in the cross-linking technique in the two products might be one of the causes of the therapeutic differences.

Even in the academic community, some popular guidelines of clinical practice have provided inconsistent and contradictory recommendations for IAHA [7,20,21,22]. In a recent meta-analysis, Campbell et al. [3] reported that IAHA is a viable option for knee OA with improvements in knee pain and function for up to 26 weeks, according to the highest level of evidence. To date, the beneficial effect of HA/cHA in a longer period remains uncertain. As the comparator with a variety of therapeutic arms [23,24,25,26], the effect of IAHA at 12 months has been variable without consistent results in comparing with the baseline. In our results, both cHA products, namely HJP and Durolane, provided significant improvements in VAS pain and stiffness scores and WOMAC total score for 52 weeks post-injection. The current consensus of IAHA is suggested for mild to moderate knee OA (K-L grade II–III) [6], as reflected in our inclusion criteria. It should be noted that the trials by Vega et al. [23] and Vaquerizo et al. [27] included the advanced stage of knee OA (K-L grade IV), which might lead to ineffective results for IAHA. Furthermore, we found that in patients with US inflammatory features at baseline examination, the percentage of inflammation significantly and monotonically decreased over 52 weeks in both groups. The effect of joint effusion before HA treatment in knee OA is inconclusive. Patients with clinical apparent effusion [28] or severe effusion [29] that indicates a severe inflammation episode are usually excluded from trials. Mild to moderate effusion has been reported to be associated with better response to IAHA [30,31]. That the proportion of all US inflammatory signs significantly decreased after IAHA in our results supports the anti-inflammatory effect of cHA. However, we acknowledge that the majority of our patients presented mild (grade I) US inflammation features. As such, we maintain that patients with severe inflammation (grade III in any US features) are not good candidates for IAHA.

All adverse events in this trial were mild following IAHA injection and were spontaneously resolved within one week. The incidences of various adverse events in both groups were comparable, and are compatible to the previous reported incidences of adverse events with cHA [7,9,10,11,27] (7.1% to 50.0%). Accordingly, our results suggest a favorable safety profile for both products.

There are some limitations in this study. First, we did not have a placebo group. The placebo arm including arthrocentesis and IA injection of saline invites ethical and methodological concerns [32]. Furthermore, Durolane’s clinical superiority over an IA placebo has already been demonstrated in patients with knee OA only [33] and its clinical effectiveness and safety for knee OA has been shown according to numerous clinical studies with a variety of comparator arms [34]. Therefore, in a head-to-head design, Durolane was chosen as a comparator rather than a placebo and our study aim was to compare the effectiveness and safety between HJP and Durolane in the treatment of knee OA. Second, our population was limited to patients with K–L grade II–III OA with no severe inflammatory signs, which is compatible with the current consensus. Nevertheless, our results cannot be generalized to all populations with knee OA, especially to those with advanced OA. Third, in the initial study design, we did not evaluate the predictor for better clinical outcomes that deserve further analyses.

Due to the biocompatibility and biodegradability of HA, it is widely applied in biomedicine in the hydrogel form [35] or as controlled release matrices [36]. Recently, HA nanoparticles [37] and HA with antioxidant nanoparticles such as cerium oxide [38] or gold [39] have been reveal as promising therapeutic approaches for knee OA. These new findings and our results support the assertion that HA is a treatment option for knee OA.

## 5. Conclusions

HJP led to better VAS pain relief than Durolane for 26 weeks post-injection. HJP provided superior improvement in terms of VAS pain and stiffness scores, WOMAC pain and stiffness scores, and satisfaction score for 39 weeks after treatment. A single injection of either HJP or Durolane is safe and effective for 52 weeks in patients with knee OA. Be that as it may, further studies are necessary to confirm the long-term effects of cHA in the treatment of knee OA.

## Figures and Tables

**Table 1:** Inclusion and exclusion criteria.

Inclusion Criteria
Age from 35 to 85 years
Radiographic Kellgren-Lawrence grade II to III
Symptoms ≥ 6 months despite conservative treatments such as analgesics, NSAIDs and/or physical therapy
Average knee pain score ≥ 30 mm on 100-mm VAS in the recent one week
Radiographic evidence of bilateral knee OA not reason for exclusion if VAS pain in contralateral knee < 30 mm
**Exclusion Criteria**
Previous orthopedic surgery in the lower extremity
Disabling osteoarthritis of hip or ankle
Previous intra-articular injection of hyaluronate within 6 months
Intra-articular injection of steroid or joint puncture within 3 months
Characteristics of severe acute synovitis under ultrasound examination, such as Grade 3 in suprapatellar synovitis [17], suprapatellar effusion [17], medial compartment synovitis or lateral compartment synovitis [18]
Any specific medical condition, such as rheumatoid arthritis, Lupus erythematous, hemiparesis, infection, neoplasm, etc., that would interfere with assessments
Confirmed or suspected pregnancy or lactating
Known allergy history to any hyaluronate product

**Table 2:** Demographic data and baseline characteristics.

	HYAJOINT Plus (*n* = 71)	Durolane (*n* = 71)	*p* Value
Age (years; mean ± SD)	66.1 ± 8.9	65.5 ± 9.7	0.722
Gender (*n*, %)			0.833
Male	15 (21.1)	13 (18.3)	
Female	56 (78.9)	58 (81.7)	
BMI (mean ± SD)	24.3 ± 3.2	24.0 ± 3.1	0.604
Body weight (*n*, %)			0.873
Normal (18.5 ≤ BMI < 24.0)	38 (53.5)	38 (53.5)	
Overweight (BMI ≥ 24.0)	23 (32.4)	21 (29.6)	
Obese (BMI ≥ 27.0)	10 (14.1)	12 (16.9)	
OA K-L grade (*n*, %)			0.387
II	30 (42.3)	24 (33.8)	
III	41 (57.7)	47 (66.2)	
Site (*n*, %)			0.861
Left	24 (33.8)	26 (36.6)	
Right	47 (66.2)	45 (63.4)	
VAS pain score (0–100; mean ± SD)	63.3 ± 12.2	60.8 ± 13.8	0.262
VAS stiffness score (0–100; mean ± SD)	43.5 ± 13.7	41.1 ± 16.2	0.345
WOMAC score (mean ± SD)	41.0 ± 14.9	38.4 ± 16.3	0.323
TUG (sec; mean ± SD)	15.3 ± 4.1	15.6 ± 3.7	0.658
SLS (sec; mean ± SD)	16.5 ± 10.3	19.7 ± 13.6	0.115
Ultrasound features (*n*, %)			
Suprapatellar synovitis	8 (11.3%)	2 (2.8%)	0.101
Suprapatellar effusion	18 (25.4%)	10 (14.1%)	0.140
Medial compartment synovitis	31 (43.7%)	28 (39.4%)	0.733
Lateral compartment synovitis	17 (23.9%)	7 (9.9%)	0.044

BMI, body mass index; OA, osteoarthritis; K–L grade, Kellgren–Lawrence grade; VAS, visual analog scale; WOMAC, Western Ontario and McMaster Universities Osteoarthritis Index; TUG, Timed “Up & Go”; SLS, single-limb stance.

**Table 3:** Comparison of VAS pain score, VAS stiffness score, WOMAC score, TUG test, and SLS test between groups.

	HYAJOINT Plus		Durolane		*p* Value ^b^	*p* Value ^c^
	Mean ± SD	*p* Value ^a^	Mean ± SD	*p* Value ^a^		
VAS pain score						0.607
Baseline	63.3 ± 12.2	-	60.8 ± 13.8	-	0.179	
4 weeks	23.3 ± 20.6	<0.001	36.0 ± 25.0	<0.001	<0.001	
12 weeks	16.6 ± 11.2	<0.001	24.5 ± 16.9	<0.001	<0.001	
26 weeks	18.1 ± 9.5	<0.001	24.4 ± 14.0	<0.001	0.003	
39 weeks	24.5 ± 10.9	<0.001	29.5 ± 12.7	<0.001	0.017	
52 weeks	35.8 ± 10.9	<0.001	36.8 ± 12.6	<0.001	0.662	
β-coefficient ^d^	−4.45		−4.50			
VAS stiffness score						0.147
Baseline	43.5 ± 13.7	-	41.1 ± 16.2	-	0.221	
4 weeks	22.6 ± 5.8	<0.001	24.4 ± 9.7	<0.001	0.212	
12 weeks	20.4 ± 2.0	<0.001	23.0 ± 6.9	<0.001	0.003	
26 weeks	29.8 ± 2.4	<0.001	31.7 ± 4.9	<0.001	0.004	
39 weeks	30.1 ± 2.8	<0.001	32.5 ± 5.1	<0.001	<0.001	
52 weeks	30.6 ± 4.1	<0.001	32.2 ± 5.0	<0.001	0.038	
β-coefficient ^d^	−0.94		−0.33			
WOMAC pain score						0.327
Baseline	10.1 ± 3.4	-	9.6 ± 4.1	-	0.253	
4 weeks	4.9 ± 4.5	<0.001	6.5 ± 4.6	<0.001	0.059	
12 weeks	1.9 ± 2.9	<0.001	3.4 ± 3.3	<0.001	0.004	
26 weeks	2.8 ± 2.0	<0.001	4.1 ± 2.9	<0.001	0.002	
39 weeks	4.1 ± 2.2	<0.001	5.1 ± 2.5	<0.001	0.013	
52 weeks	5.4 ± 2.2	<0.001	5.6 ± 2.5	<0.001	0.509	
β-coefficient ^d^	−0.87		−0.75			
WOMAC stiffness score						0.013
Baseline	1.8 ± 1.8	-	1.3 ± 1.7	-	0.028	
4 weeks	1.5 ± 1.0	0.017	1.5 ± 1.2	0.102	0.694	
12 weeks	1.1 ± 0.4	<0.001	1.4 ± 0.9	0.374	0.003	
26 weeks	1.0 ± 0.3	<0.001	1.3 ± 0.8	0.705	0.007	
39 weeks	1.1 ± 0.6	0.001	1.4 ± 0.9	0.476	0.034	
52 weeks	1.2 ± 0.5	0.002	1.4 ± 0.8	0.436	0.039	
β-coefficient ^d^	−0.12		0.01			
WOMAC function score						0.933
Baseline	29.0 ± 11.2	-	27.5 ± 12.0	-	0.268	
4 weeks	18.0 ± 11.8	<0.001	21.8 ± 12.3	<0.001	0.077	
12 weeks	13.6 ± 9.1	<0.001	19.1 ± 10.9	<0.001	0.001	
26 weeks	16.1 ± 8.2	<0.001	18.6 ± 8.8	<0.001	0.069	
39 weeks	21.9 ± 9.0	<0.001	22.0 ± 8.3	<0.001	0.900	
52 weeks	26.3 ± 8.0	0.049	25.4 ± 7.8	0.124	0.461	
β-coefficient ^d^	−0.43		−0.42			
WOMAC total score						0.530
Baseline	41.0 ± 14.9	-	38.4 ± 16.3	-	0.174	
4 weeks	24.4 ± 16.2	<0.001	29.8 ± 16.9	<0.001	0.064	
12 weeks	16.6 ± 11.8	<0.001	23.9 ± 14.2	<0.001	<0.001	
26 weeks	20.0 ± 9.3	<0.001	24.1 ± 11.4	<0.001	0.018	
39 weeks	27.1 ± 10.4	<0.001	28.5 ± 10.6	<0.001	0.635	
52 weeks	32.9 ± 9.4	<0.001	32.4 ± 10.2	0.002	0.778	
β-coefficient ^d^	−1.46		−1.16			
TUG time (sec)						0.091
Baseline	15.3 ± 4.1	-	15.6 ± 3.7	-	0.707	
4 weeks	14.5 ± 3.2	0.001	16.2 ± 3.5	0.010	0.002	
12 weeks	14.6 ± 3.3	0.009	17.0 ± 3.9	<0.001	<0.001	
26 weeks	14.6 ± 3.5	0.075	17.0 ± 3.9	<0.001	<0.001	
39 weeks	15.0 ± 4.0	0.513	17.2 ± 4.0	<0.001	<0.001	
52 weeks	15.4 ± 4.0	0.782	16.8 ± 3.9	0.003	0.018	
β-coefficient ^d^	0.06		0.26			
SLS time (sec)						0.011
Baseline	16.5 ± 10.3	-	19.7 ± 13.6	-	0.061	
4 weeks	20.4 ± 14.2	0.001	20.3 ± 13.6	0.225	0.910	
12 weeks	22.6 ± 17.1	<0.001	19.9 ± 14.1	0.787	0.321	
26 weeks	24.4 ± 20.7	<0.001	21.9 ± 18.0	0.042	0.450	
39 weeks	26.0 ± 22.9	<0.001	22.3 ± 18.1	0.019	0.264	
52 weeks	27.8 ± 26.8	<0.001	23.3 ± 18.0	0.002	0.215	
β-coefficient ^d^	2.25		0.72			

*p* value ^a^, comparison of the various outcome variables at each follow-up point in time with those at the baseline within the group using linear regression models with the generalized estimation equation (GEE) method; *p* value ^b^, comparison of the variables between groups using linear regression with adjustment for age, gender, body weight, and osteoarthritis Kellgren–Lawrence (OA KL) grade; *p* value ^c^, examination of interaction of the study group with time using linear regression models with the GEE model with adjustment for age, gender, body weight, and OA KL grade; β-coefficient ^d^, regression coefficient indicating time trend.

**Table 4:** Comparison of satisfaction score between groups.

Satisfaction Score (mm)	HYAJOINT Plus	Durolane	*p* Value
4 weeks	76.8 ± 13.8	65.9 ± 15.8	<0.001
12 weeks	88.9 ± 7.1	81.4 ± 11.8	<0.001
26 weeks	92.3 ± 7.1	84.9 ± 12.1	<0.001
39 weeks	76.6 ± 7.2	72.1 ± 10.1	<0.001
52 weeks	73.8 ± 8.6	72.5 ± 10.7	0.464

*p* value, the between-group difference was tested using independent *t*-tests.

**Table 5:** Adverse events reported in the study groups.

	HYAJOINT Plus (*n* = 71)	Durolane (*n* = 71)	*p* Value
	Patient No. (%)	Patient No. (%)	
*Related events*			
Injection site pain	8 (11.3%)	11 (15.5%)	0.622
Joint swelling	4 (5.6%)	5 (7.0%)	1.000
Joint stiffness	5 (7.0%)	7 (9.9%)	0.763
Joint soreness	0 (0.0%)	1 (1.4%)	1.000
Foreign body sensation	6 (8.5%)	10 (14.1%)	0.426
Total number	23 (32.4%)	34 (47.9%)	0.087
*Unrelated events*			
Infection	0 (0.0%)	1 (1.4%)	1.000

Patients are counted once for each unique adverse event and may have had >1 unique adverse event. The *p* value, between-group difference was tested using the Chi-square test.

## Data Availability

The datasets used during the current study are available from the corresponding author on reasonable request.

## Supplementary Materials

The following supporting information can be downloaded at: https://www.mdpi.com/article/10.3390/pharmaceutics14091783/s1, Figure S1: Schematic representation of cross-linking of hyaluronate with 1,4-butanediol diglycidyl ether (BDDE); Table S1: Comparison of ultrasound inflammatory features between groups.
