# Supplementary material for: Single Injection of Cross-Linked Hyaluronate in Knee Osteoarthritis: A 52-Week Double-Blind Randomized Controlled Trial
Source: Pharmaceutics. 2022 Aug 25;14(9):1783. doi: 10.3390/pharmaceutics14091783 (PMC9504468; doi:10.3390/pharmaceutics14091783)
Supplement: Supplementary file 1 [file pharmaceutics-14-01783-s001.zip › Suppl Figure S1.pdf]

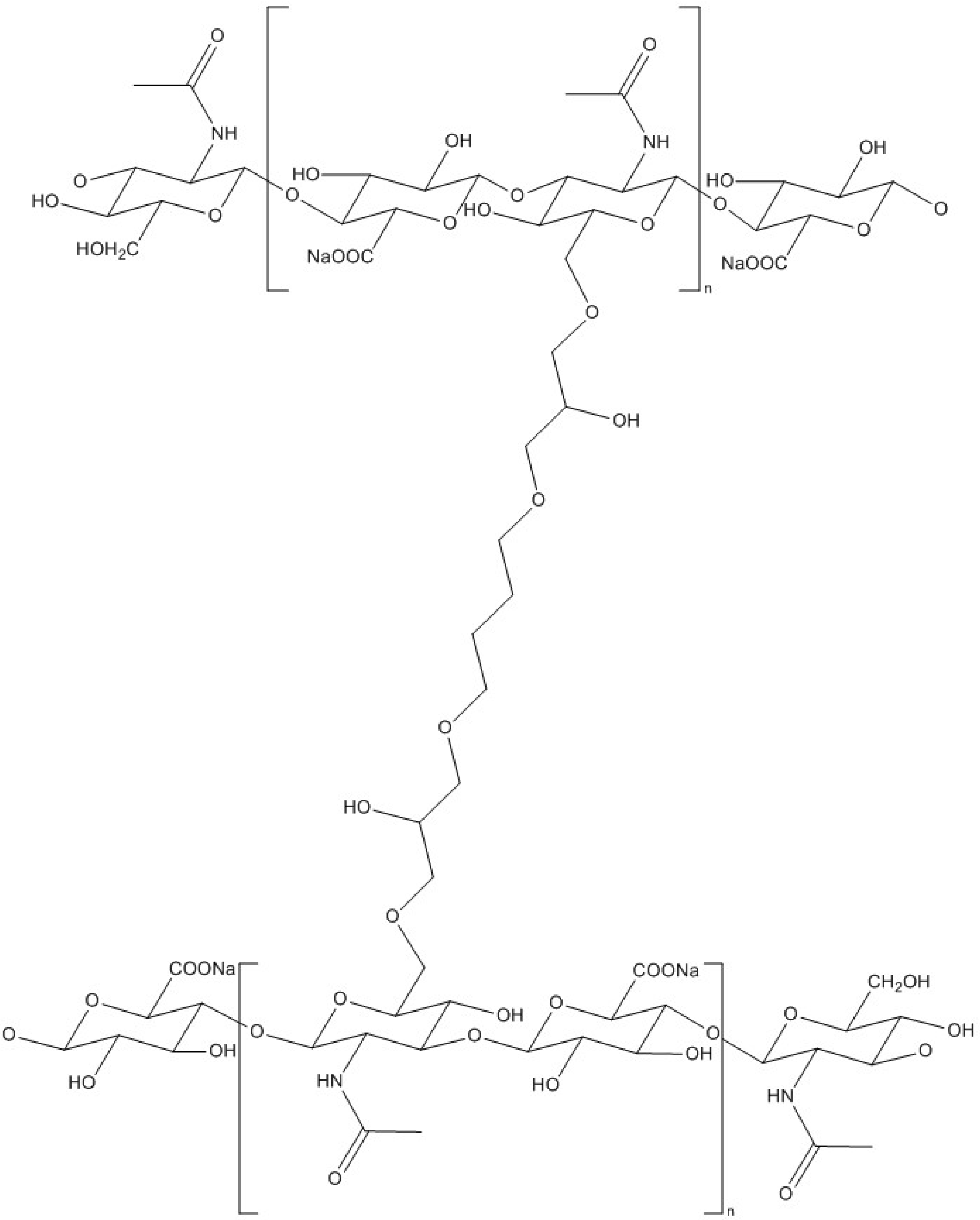

Supplementary Figure S1. Schematic representation of Cross-linking of hyaluronate with 1,4-butanediol diglycidyl ether (BDDE). Both HYAJOINT Plus and Durolane are produced by biofermentation and cross-linked by BDDE. HYAJOINT Plus is synthesized using Crosslinked Hyaluronic Acid Platform (CHAP) technique that uses two different temperatures and longtime of reaction to ensure the completeness of crosslinking reaction. Durolane is cross-linked using the non-animal stabilized hyaluronic acid (NASHA) system that increases the hyaluronate reaction concentration during the cross-linking reaction under one reaction temperature.
