# Supplementary material for: Single Injection of Cross-Linked Hyaluronate in Knee Osteoarthritis: A 52-Week Double-Blind Randomized Controlled Trial
Source: Pharmaceutics. 2022 Aug 25;14(9):1783. doi: 10.3390/pharmaceutics14091783 (PMC9504468; doi:10.3390/pharmaceutics14091783)
Supplement: Supplementary file 1 [file pharmaceutics-14-01783-s001.zip › Supplementary Table S1.pdf]

Supplementary Table S1. Comparison of ultrasound inflammatory features between groups.

**Ultrasound features**	**HYAJOINT Plus**	**Durolane**	Remark
Suprapatellar synovitis (SPS), n (%)			Grade I SPS in all cases
Baseline	8	2	
4 weeks	4 (50.0%)	0 (0.0%)	
12 weeks	0 (0.0%)[b]	0 (0.0%)	
26 weeks	1 (16.7%)[b]	0 (0.0%)	
39 weeks	1 (16.7%)[b]	0 (0.0%)	
52 weeks	0 (0.0%)[b]	0 (0.0%)[a]	
p value	<0.001	0.026	
Suprapatellar effusion (SPE), n (%)			Grade I SPE in other cases
Baseline	18 (Gr. II: 1)	10 (Gr. II: 1)	
4 weeks	9 (50.0%)	4 (40.0%)	
12 weeks	4 (23.5%)[a]	3 (30.0%)	
26 weeks	2 (11.8%)[a]	0 (0.0%)	
39 weeks	4 (25.0%)[b]	2 (25.0%)[b]	
52 weeks	8 (53.3%)[c]	3 (37.5%)[b]	
p value	<0.001	0.002	
Medial compartment synovitis (MCS), n (%)			Grade I MCS in all cases
Baseline	31	28	
4 weeks	21 (67.7%)	21 (75.0%)	
12 weeks	14 (46.7%)[a]	10 (50.0%)[a]	
26 weeks	11 (37.9%)[b]	7 (26.9%)[b]	
39 weeks	13 (44.8%)[b]	8 (36.4%)[f]	
52 weeks	14 (48.3%)[b]	6 (26.1%)[e]	
p value	<0.001	<0.001	
Lateral compartment synovitis (LCS), n (%)			Grade I LCS in all cases
Baseline	17	7	
4 weeks	9 (52.9%)	4 (57.1%)	
12 weeks	7 (43.8%)[a]	1 (14.3%)	
26 weeks	5 (33.3%)[b]	2 (28.6%)	
39 weeks	5 (33.3%)[b]	0 (0.0%)[b]	
52 weeks	5 (33.3%)[b]	1 (20.0%)[b]	
p value	<0.001	<0.001	

p value, the linear trend in percentage within the group was tested using the Cochran-Armitage trend test

[a]: one case missing; [b]: two cases missing; [c]: three cases missing; [d]: four cases missing;
[e]: five cases missing; [f]: six cases missing
